# Filter-free exhaustive odds ratio-based genome-wide interaction approach pinpoints evidence for interaction in the HLA region in psoriasis

**DOI:** 10.1186/s12863-015-0174-3

**Published:** 2015-02-06

**Authors:** Laura Grange, Jean-François Bureau, Iryna Nikolayeva, Richard Paul, Kristel Van Steen, Benno Schwikowski, Anavaj Sakuntabhai

**Affiliations:** Department of Genomes and Genetics, Institut Pasteur, Functional Genetics of Infectious Diseases Unit, Paris, 75015 France; CNRS URA3012, Paris, 75015 France; Université Paris Diderot, Paris, 75013 France; Department of Genomes and Genetics, Institut Pasteur, Systems Biology Lab, Paris, 75015 France; Université Paris-Descartes, Sorbonne Paris Cité, Paris, France; Systems and Modeling Unit, Montefiore institute, University of Liège, Liège, Belgium; Bioinformatics and Modeling, GiGA-R, University of Liège, Liège, Belgium

**Keywords:** Genome-wide interaction studies, Epistasis, Plink, MBMDR, IOR

## Abstract

**Background:**

Deciphering the genetic architecture of complex traits is still a major challenge for human genetics. In most cases, genome-wide association studies have only partially explained the heritability of traits and diseases. Epistasis, one potentially important cause of this missing heritability, is difficult to explore at the genome-wide level. Here, we develop and assess a tool based on interactive odds ratios (I_OR_), Fast Odds Ratio-based sCan for Epistasis (FORCE), as a novel approach for exhaustive genome-wide epistasis search. I_OR_ is the ratio between the multiplicative term of the odds ratio (OR) of having each variant over the OR of having both of them. By definition, an I_OR_ that significantly deviates from 1 suggests the occurrence of an interaction (epistasis). As the I_OR_ is fast to calculate, we used the I_OR_ to rank and select pairs of interacting polymorphisms for P value estimation, which is more time consuming.

**Results:**

FORCE displayed power and accuracy similar to existing parametric and non-parametric methods, and is fast enough to complete a filter-free genome-wide epistasis search in a few days on a standard computer. Analysis of psoriasis data uncovered novel epistatic interactions in the HLA region, corroborating the known major and complex role of the HLA region in psoriasis susceptibility.

**Conclusions:**

Our systematic study revealed the ability of FORCE to uncover novel interactions, highlighted the importance of exhaustiveness, as well as its specificity for certain types of interactions that were not detected by existing approaches. We therefore believe that FORCE is a valuable new tool for decoding the genetic basis of complex diseases.

**Electronic supplementary material:**

The online version of this article (doi:10.1186/s12863-015-0174-3) contains supplementary material, which is available to authorized users.

## Background

During the past decade, many genome-wide association studies (GWAS) have aimed to identify new genetic factors determining susceptibility to a variety of diseases [1,2]. Although promising and sometimes successful, these large-scale studies have only led to modest advances [3]. One explanation is that the underlying model that single SNPs contribute independently to the complex trait may frequently be too simple. Rather, complex traits are likely to result from a complex interplay between genes, notably epistatic gene-environment and gene-gene interactions [4].

The principal obstacles in a genome-wide search for epistasis are statistical power to overcome the limitations of multiple testing and the computational time of the search itself. Over the past decades, many tools have been developed for epistasis detection using various statistical methods [5,6], including those based on regression [7-11], linkage disequilibrium and haplotypes [12,13], and Bayesian approaches [14,15]. Alternative approaches are based on data-filtering, machine-learning and data mining [16-19]. Here, we present an approach that detects pairwise epistasis on a genome-wide scale based on the classical interaction odds ratio (I_OR_). Introduced by Piegorsch et al. in 1994 [20], this approach has mainly been used for the detection of gene-environment interactions in case-only designs [21]. VanderWeele et al. [22] showed how the use of I_OR_ can help reveal mechanistic interactions in case-only datasets.

Firstly, we report on the first efficient implementation of an approach for genome-wide epistasis detection, which we call FORCE (Fast Odds Ratio sCan for Epistasis). Due to its mathematical simplicity, the approach is suitable for exhaustive unfiltered epistasis analysis; i.e., the exact value of the I_OR_ statistic can be evaluated for all pairs of genotyped SNPs in reasonable time on a standard computer. We introduce the mathematics to compute exact P-values for the most extreme values of I_OR_.

Secondly, we describe the application of FORCE to the Welcome Trust Case Control Consortium (WTCCC) data on psoriasis, and analyze the previously unknown statistical interactions we found in the light of already-known results.

Lastly we ask whether the statistical interactions detected by FORCE were found due to its exhaustiveness and/or its underlying genetic model, and we present evidence for both. We show that the restriction of FORCE to analyzing only certain SNPs selected according to their marginal effect on psoriasis (as previously described by Knight et al. [23]) strongly limits the statistical significance of the results. We then benchmark the performance of FORCE and other popular methods to detect simulated epistatic interactions, always using exhaustive search. Under different common models for interaction and noise, FORCE consistently detects certain types of interactions better than other approaches.

## Methods

### Definition of interaction odds ratio (I_OR_)

For any given pair of SNPs, the interaction odds-ratio statistic I_OR_ is calculated from a pair of 2×2 contingency tables. These tables are derived from 3×3 tables of all allele combinations, by collapsing them according to a dominant or recessive model (see Table 1). Following preliminary evidence that the dominant model allowed more efficient detection of epistasis (Table 2), all analyses were performed using this dominant genetic model.

**Table 1:** **Contingency table under a dominant model**

**SNP1**	**SNP2**	**Cases**	**Controls**
AA	BB	*α*	*β*
AA	Bb or bb	*γ*	*δ*
Aa or aa	BB	*ε*	*ζ*
Aa or aa	Bb or bb	*η*	*θ*

Major alleles are respectively A and B for each SNP and minor alleles a and b. The risk allele is assumed to be the minor allele.

**Table 2:** **Power and Family-wise error rate (FWER) for detection of the functional pair using a dominant or recessive transmission assumption in 6 different epistasis models**

**Genetic model**	**Test**	**Model 1**	**Model 2**	**Model 3**	**Model 4**	**Model 5**	**Model 6**
**Dominant**	Power	1	0.97	0.96	1	0.93	0.99
	FWER	0.05	0.02	0.02	0.06	0.05	0.04
**Recessive**	Power	0.93	0.96	0.01	0.01	0	0
	FWER	0.04	0.07	0.02	0.02	0.03	0.01

We define the following odds ratios:

$$ {\mathrm{OR}}_1=\frac{\beta \varepsilon }{\alpha \zeta },\ {\mathrm{OR}}_2=\frac{\beta \gamma }{\alpha \delta },{\mathrm{OR}}_{1*2}=\frac{\beta \eta }{\alpha \theta },\ \mathrm{and}\ {\mathrm{I}}_{\mathrm{OR}}=\frac{{\mathrm{OR}}_{1*2}}{{\mathrm{OR}}_1\cdot {\mathrm{OR}}_2}=\frac{\alpha \delta \zeta \eta}{\beta \gamma \varepsilon \theta}. $$

Note that I_OR_ is undefined when the denominator of this expression becomes zero. For formal consistency, we therefore added a pseudocount of 1 to each cell of the two contingency tables.

### Statistical significance: Empirical and exact P-values

Note that an I_OR_ of *x* equals an I_OR_ of 1/*x* after exchanging counts between cases and controls. We define universal I_OR_, u(I_OR_):

$$ \mathrm{u}\left({\mathrm{I}}_{\mathrm{OR}}\right)=\frac{1}{{\mathrm{I}}_{\mathrm{OR}}}\mathrm{if}\ {\mathrm{I}}_{\mathrm{OR}}\le\ 1\ \mathrm{and}\ \mathrm{u}\left({\mathrm{I}}_{\mathrm{OR}}\right)={\mathrm{I}}_{\mathrm{OR}}\ \mathrm{if}\ {\mathrm{I}}_{\mathrm{OR}}>1. $$

This definition allows us to express significant deviations of u(I_OR_) from the expectation of 1 using a one-tailed P-value.

Pairs with high u(I_OR_) were identified by the straightforward algorithm that computes u(I_OR_) for each pair of given SNPs. Our C implementation encodes, in a preprocessing step, all data related to any given SNP into a bit string, and then uses fast logical and bit-counting functions to compute u(I_OR_) for all pairs.

Marginal empirical P-values for any given pair of SNPs were calculated as the proportion of u(I_OR_) values from randomly generated permutations of case–control labels that were larger than or equal to the value of u(I_OR_) obtained for the same pair in real data. The number of permutations performed (1000 for simulated data, 100,000 for real data) was adapted to the number of tests performed in these two scenarios.

Exact P-values were calculated using

$$ p= Pr\left(\mathrm{u}\left({\mathrm{I}}_{\mathrm{OR}}\right)\ge \mathrm{x}\right)=2\cdot \sum_{\begin{array}{l}\left(\alpha \hbox{'},\gamma \hbox{'},\varepsilon \hbox{'},\eta \hbox{'}\right):\ {I}_{OR}\ge \mathrm{x},\\ {}\alpha \hbox{'}+\gamma \hbox{'}+\varepsilon \hbox{'}+\eta \hbox{'}=\alpha +\gamma +\varepsilon +\eta \end{array}}\frac{\left(\underset{\alpha \hbox{'}}{\alpha +\beta}\right)\left(\underset{\gamma \hbox{'}}{\gamma +\delta}\right)\left(\underset{\varepsilon \hbox{'}}{\varepsilon +\zeta}\right)\left(\underset{\eta \hbox{'}}{\eta +\theta}\right)}{\left(\underset{\alpha \hbox{'}+\gamma \hbox{'}+\varepsilon \hbox{'}+\eta \hbox{'}}{\alpha +\beta +\gamma +\delta +\varepsilon +\zeta +\eta +\theta}\right)} $$

and computed by the straightforward algorithm with four nested loops to cover all required parameter tuples (α’,γ’,ε’,η’). Each inner loop only visits those parameter values that correspond to possible tuples with α’ + γ’ + ε’ + η’ = α + γ + ε + η, given the parameter values in the outer loop. Summed are those terms with u(I_OR_) ≥ x.

### Application of FORCE to psoriasis data

To evaluate FORCE, we assessed its performance on the WTCCC psoriasis dataset. Initial GWAS and further analyses performed on these data are described in [24]. Following general practice for pre-processing, we excluded potentially low-quality SNP data from further analysis. Specifically, we discarded i) any individual whose total missing rate was above 0.05, ii) any SNP with a frequency of missing data above 0.05, and iii) any SNP with minor allele frequency below 0.05. After pre-processing, our dataset consisted of 2,618 cases, 2,737 controls and 491,191 SNPs, corresponding to approximately 1.2 × 10^11^ SNP pairs. We excluded pairs with a genomic distance of less than 100 kb to avoid pairs in linkage disequilibrium. In addition, we found that low row and cell counts in the contingency table (Table 1) can lead to extreme but frequently not significant values of u(I_OR_). For the purposes of this study, we excluded 3,521,114 SNP pairs with a total count of less than 50 in any row, or less than 5 in any cell of the contingency table. In addition to FORCE, we performed PLINK (FastEpistasis mode) on the top-ranked 500 pairs to compare the results obtained with both methods.

### Comparison of exhaustive FORCE with semi-exhaustive and conditional search

To assess the utility of exhaustive search, we constructed a reference dataset of SNPs previously implicated in psoriasis. We started with a set of 34 SNPs from two previous reviews on psoriasis genetics [25,26] that were part of our psoriasis dataset. After applying quality control thresholds (described above), 18 SNPs remained.

Following general practice for genome-wide approaches, for exhaustive and semi-exhaustive searches, we used a genome-wide significance threshold of $\mathrm{p}=\frac{2\times 0.05}{{\left({10}^6\right)}^2}={10}^{-13}$, which is based on a model of the human genome with 10^6^ independent SNPs [27].

### Comparison of FORCE with other approaches on simulated datasets

We simulated datasets of 10 biallelic SNPs over 200 cases and 200 controls following the Hardy-Weinberg equilibrium model. Interactions were simulated according to six different previously described models without main effect [28] (Table 3). These models represent pure epistasis effects, and not confounding main effects. Model 1 is an interaction effect in which high risk of disease occurs when inheriting heterozygous genotypes at either locus (Aa or Bb) but not both. Model 2 represents high risk of disease when inheriting two high-risk alleles that could be A and/or B. Models 3–6 correspond to the epistasis model discovery method described by Moore et al. [29]. Each of these models represents an interaction effect without any main effects. Allele frequencies are p = 0.25 and q = 0.75 for model 3 and 4, p = 0.1 and q = 0.9 for model 5 and 6.

**Table 3:** **Penetrances and allele frequencies (p,q) used to simulate the interaction models – from Ritchie** [28]

**Model 1**				**Model 2**				**Model 3**			
	**BB**	**Bb**	**bb**		**BB**	**Bb**	**bb**		**BB**	**Bb**	**bb**
AA	0	0.10	0	AA	0	0	0.10	AA	0.08	0.07	0.05
Aa	0.10	0	0.10	Aa	0	0.05	0	Aa	0.10	0	0.10
aa	0	0.10	0	aa	0.10	0	0	aa	0.03	0.10	0.04
p = 0.5, q = 0.5				p = 0.5, q = 0.5				p = 0.25, q = 0.75			
**Model 4**				**Model 5**				**Model 6**			
	**BB**	**Bb**	**bb**		**BB**	**Bb**	**bb**		**BB**	**Bb**	**bb**
AA	0	0.01	0.09	AA	0.07	0.05	0.02	AA	0.09	0.001	0.02
Aa	0.04	0.01	0.08	Aa	0.05	0.09	0.01	Aa	0.08	0.07	0.005
aa	0.07	0.09	0.03	aa	0.02	0.01	0.03	aa	0.003	0.007	0.02
p = 0.25, q = 0.75				p = 0.10, q = 0.9				p = 0.10, q = 0.9			

Marginal penetrances for each genotype are identical as we simulate pure epistasis effects.

For each of the six models, we generated 100 datasets in each of the 16 conditions of the presence or absence of four of the most commonly encountered sources of noise: missing data (MS), genotyping errors (GE), genetic heterogeneity (GH), and phenocopy (PC).

For GH, two independent interactions were simulated instead of one, each interaction being risk-associated in half of the affected cases. When PC was simulated, interaction affected the trait for half of the cases, emulating an unknown environmental effect. GE and MS were simulated at 5%, as previously described [28].

An epistatic pair of SNPs was considered as detected if the empirical P-value was below 0.001, i.e., below 0.05 after Bonferroni correction. Power was estimated as *n*/100, where *n* is the number of datasets with detection(s). When two pairs (P_1_, P_2_) of SNPs were simulated, detection was counted under one of three different conditions: D1) when P_1_ and P_2_ were detected, D2) when P_1_ was detected, or D3) when P_1_ or P_2_ was detected. Family-wise error rate (FWER) was calculated as *m*/100, where *m* is the number of datasets for which at least one pair other than the simulated pair was detected.

## Results

### FORCE enables exhaustive unfiltered epistasis analysis

The FORCE method for epistasis detection is based on the choice of a dominant or recessive model that collapses combinations of allele counts into two 2×2 incidence tables (see Methods). Interactions are then detected as extreme values of the I_OR_ statistic. We implemented the FORCE method for epistasis in C language [30]. Due to its mathematical simplicity and efficient implementation, the computation of I_OR_ could be performed rapidly, compared to other approaches (4.3 days on a single core of a standard computer). Table 4 shows running times of different methods selected for this study.

**Table 4:** **Average time needed to exhaustively test one/all 1.25×10** ^**11**^ **pairs among 500,000 SNPs using a single-core CPU computer**

**Software**	**Time for one/all SNPs (single core)**
MB-MDR [16]	5×10-3 s/20 years [31]
PLINK Epistasis [7]	2×10-4 s/289 days [5]
PLINK FastEpistasis [8]	2×10-5 s/29 days [32]
FORCE	3×10-6 s/4.3 days
GWIS - 3 filters [33]	1.6×10-6 s/2.2 days [33]
GWIS - 1 filter [33]	3.8×10-7 s/0.5 days [33]

We included the recent GWIS approach that is described as ‘exhaustive’, but uses filtering to avoid computing test statistics for all pairs of SNPs.

### Identification of statistically strong interactions requires exhaustive search

To assess the value of exhaustive search, we first evaluated the performance of a conventional, non-exhaustive approach of constraining the analysis to pairs of SNPs that were previously shown to have main effects associated with the phenotype. We therefore performed a constrained analysis on all pairs of 18 high-quality SNPs that had main effects on psoriasis in previous GWA studies (see Methods). Table 5 gives the best 25 hits obtained through this approach when evaluated on the WTCCC dataset on psoriasis [24] (the results of all pairs are shown in Additional file 1: Table S1). None of the 153 pairs reached a significant interaction P-value below a genome-wide significance threshold of 10^−13^.

**Table 5:** **Results from conditional search, restricted to pairs of previously implicated SNPs**

**First GWAS SNP**		**Second GWAS SNP**		**FORCE**		**PLINK FastEpistasis**
**rs number**	**Chromosomal location**	**rs number**	**Chromosomal location**	**I**_**OR**_	**Empirical p-value**	**p-value**
rs10484554	6p21.33	rs27524	5q15	6.846	0.008882	0.003095
rs10484554	6p21.33	rs3134792	6p21.33	1.068	0.3014	0.007746
rs2201841	1p31.3	rs3213094	5q33.3	4.737	0.02952	0.012373
rs3134792	6p21.33	rs4795067	17q11	3.188	0.07419	0.012783
rs20541	5q31	rs17716942	2q24	6.987	0.008212	0.013389
rs702873	2p16	rs4795067	17q11	3.414	0.06466	0.014129
rs10484554	6p21.33	rs4795067	17q11	2.597	0.1071	0.018096
rs610604	6q23	rs17716942	2q24	6.591	0.01025	0.023261
rs3213094	5q33.3	rs12580100	12q13.2	5.132	0.02349	0.028993
rs4649203	1p36	rs240993	6q21	2.270	0.1319	0.037791
rs4649203	1p36	rs702873	2p16	1.237	0.266	0.041136
rs3134792	6p21.33	rs27524	5q15	11.840	0.000581	0.041483
rs702873	2p16	rs2546890	5q33.3	0.804	0.37	0.041729
rs27524	5q15	rs17716942	2q24	5.280	0.02158	0.045812
rs610604	6q23	rs6701216	1q21	4.289	0.03837	0.057701
rs2201841	1p31.3	rs2546890	5q33.3	2.587	0.1077	0.059206
rs27524	5q15	rs7993214	13q14.11	3.596	0.05793	0.059609
rs3134792	6p21.33	rs3213094	5q33.3	2.610	0.1062	0.072999
rs702873	2p16	rs2201841	1p31.3	1.669	0.1964	0.083717
rs10484554	6p21.33	rs12580100	12q13.2	2.535	0.1113	0.086631
rs4649203	1p36	rs6701216	1q21	3.518	0.06072	0.086671
rs2201841	1p31.3	rs27524	5q15	1.666	0.1968	0.088785
rs4112788	1q21.3	rs7993214	13q14.11	1.546	0.2137	0.090419
rs240993	6q21	rs7993214	13q14.11	1.896	0.1685	0.096038
rs6701216	1q21	rs8016947	14q13	1.087	0.2971	0.100508

A more comprehensive approach, to which we will here refer to as *semi-exhaustive*, constrains only one of the SNPs in a pair to a set of previously identified SNPs [8]. Table 6 shows, for each of the 18 previously identified “main effects” SNPs, the highest-scoring interactors, according to the FORCE and PLINK FastEpistasis statistics. Note that FORCE and PLINK identified a few genome-wide significant interactions with P-values as low as 10^−20^.

**Table 6:** **Semi-exhaustive search among SNP pairs containing a GWAS-identified SNP**

**GWAS-identified SNP**				**Highest-scoring interactor with GWAS-identified SNP**							
				**FORCE u(I**_**OR**_ **)**					**PLINK FastEpistasis Z-score**		
**rs number**	**Chromosomal location**	**Risk allele OR**	**Single association p-value**	**rs number**	**Chromosomal location**	**u(I**_**OR**_ **)**	**Empirical p-value**	**Exact p-value** ^**a**^	**rs number**	**Chromosomal location**	**Exact p-value**
rs10484554	6p21.33	4.66	4.0E-214	rs4151664	6p21.33	2.97	<10E-06	7.86E-10	rs28615950	6p21.3	**2.12E-14**
rs2546890	5q33.3	1.54	1.0E-20	rs7525345	1p31.1	2.53	7.10E-04	2.17E-06	rs4796093	17q12	1.24E-06
rs6701216	1q21	1.45	6.2E-05	rs2156892	22q11.22	2.5	<10E-06	1.30E-13	rs10853580	18q21.1	4.99E-07
rs4112788	1q21.3	1.41	6.5E-09	No pair meeting all inclusion criteria					rs4459983	4q21.1	3.35E-08
rs7993214	13q14.11	1.41	2.0E-06	No pair meeting all inclusion criteria					rs10800559	1q23.3	4.04E-08
rs3213094	5q33.3	1.39	5.0E-11	No pair meeting all inclusion criteria					rs10512686	5p13.1	8.12E-06
rs17716942	2q24	1.29	1.1E-13	rs16928722	10q22.1	2.69	4.10E-06	2.78E-06	rs2553680	8q13.2	5.67E-07
rs20541	5q31.1	1.27	5.0E-15	No pair meeting all inclusion criteria					rs17171818	5q31.2	5.63E-07
rs240993	6q21	1.25	5.3E-20	rs4727157	7q21.12	2.78	<10E-06	**1.88E-20**	rs2877327	22q12.1	1.26E-07
rs4795067	17q11	1.19	4.0E-11	No pair meeting all inclusion criteria					rs3819847	3q27.3	4.59E-07
rs8016947	14q13	1.19	1.5E-11	No pair meeting all inclusion criteria					rs11071746	15q22.31	1.58E-08
rs610604	6q23	1.19	7.0E-07	rs17585537	3p26.2	2.69	<10E-06	**6.47E-19**	rs4794888	17q11.1	1.11E-06
rs12580100	12q13.2	1.17	1.0E-06	rs7565742	2q31.2	3.39	<10E-06	**6.80E-20**	rs2992154	13q21.31	2.07E-06
rs4649203	1p36	1.13	6.8E-08	No pair meeting all inclusion criteria					rs7661684	4q28.1	1.70E-06
rs2201841	1p31.3	1.13	3.0E-08	No pair meeting all inclusion criteria					rs12783252	10q26.11	3.79E-06
rs27524	5q15	1.13	2.6E-11	No pair meeting all inclusion criteria					rs7849719	9q21.31	1.37E-08
rs702873	2p16	1.12	3.6E-09	No pair meeting all inclusion criteria					rs10897897	11q13.4	1.79E-06
rs3134792	6p21.33	NR	1.0E-09	rs1062070	6p21.32	2.88	<10E-06	2.85E-10	rs1062070	6p21.32	**5.25E-14**

^a^Bold data are genome-wide significant interactions.

Finally, the relatively low computational complexity required for the FORCE statistic allowed us to perform exhaustive analysis of all SNP pairs in the psoriasis dataset. The results are shown in Table 7 (100 best hits shown in Additional file 1: Table S2). Strikingly, the best resulting P-values are another 20 orders of magnitude lower than the P-values identified by semi-exhaustive search. This shows that a large number of the most significant interactions are missed by the semi-exhaustive approach, and hence that the possibility of discovering the statistically best-supported interactions requires an exhaustive approach. Interestingly, FORCE and PLINK identify distinct interactions.

**Table 7:** **FORCE Exhaustive search top hits, and PLINK FastEpistasis results in WTCCC psoriasis data**

**SNP pair description**				**Epistasis search results**		
**First SNP**		**Second SNP**		**FORCE**		**PLINK FastEpistasis**
**rs number**	**Chromosomal location (position)**	**rs number**	**Chromosomal location (position)**	**u(I**_**OR**_ **)**	**p-value** ^**a**^	**p-value** ^**a**^
rs4151664	6p21.33 (31,920,873)	rs9267532	6p21.33 (31,639,979)	10.588	**3.32E-33**	**4.65E-33**
rs4151664	6p21.33 (31,920,873)	rs2227956	6p21.33 (31,778,272)	9.662	**2.02E-26**	7.72E-06
rs3132468	6p21.33 (31,475,486)	rs4151664	6p21.33 (31,920,873)	9.571	**3.14E-25**	1.82E-07
rs9267546	6p21.33 (31,673,436)	rs4151664	6p21.33 (31,920,873)	8.340	**1.08E-31**	**2.88E-31**
rs4151664	6p21.33 (31,920,873)	rs2260000	6p21.33 (31,593,476)	7.749	**3.74E-18**	1.81E-06
rs2523608	6p21.33 (31,322,559)	rs4151664	6p21.33 (31,920,873)	7.695	**1.08E-18**	4.93E-09
rs4151664	6p21.33 (31,920,873)	rs2855807	6p21.33 (31,469,323)	7.444	**3.88E-17**	3.35E-05
rs2596464	6p21.33 (31,416,156)	rs4151664	6p21.33 (31,920,873)	7.379	**2.67E-15**	5.40E-10
rs3129939	6p21.32 (31,412,961)	rs3131296	6p21.32 (32,172,993)	7.376	**6.43E-41**	**4.45E-30**
rs2516464	6p21.33 (31,416,156)	rs12663103	6p21.32 (32,161,324)	7.229	4.25E-13	3.74E-07
rs6906662	6p21.32 (32,266,506)	rs9267649	6p21.33 (31,824,828)	7.187	**1.59E-25**	2.86E-06
rs12153855	6p21.33 (32,074,804)	rs2523608	6p21.33 (31,322,559)	7.181	**1.59E-23**	6.91E-09
rs4149013	12p12.2 (21,282,410)	rs9356206	6q27 (164,818,834)	6.485	9.82E-09	1.11E-05
rs535586	6p21.33 (31,860,337)	rs2523589	6p21.33 (31,327,334)	6.299	**1.84E-44**	**5.45E-43**
rs2523589	6p21.33 (31,327,334)	rs659445	6p21.33 (31,864,304)	6.268	**4.08E-45**	**9.51E-44**
rs408359	6p21.32 (32,141,883)	rs4151664	6p21.33 (31,920,873)	6.038	**4.30E-21**	**1.64E-21**
rs2164182	chr11q21 (95,981,029)	rs16864296	1q24.3 (171,236,326)	5.945	8.34E-08	9.86E-06
rs2227956	6p21.32 (31,778,272)	rs2523589	6p21.33 (31,327,334)	5.851	**1.39E-42**	**2.91E-42**
rs12050395	14q31.3 (86,210,504)	rs2301092	5q14.3 (83,363,112)	5.831	1.67E-08	2.33E-06
rs12663103	6p21.32 (32,161,324)	rs9267649	6p21.33 (31,824,828)	5.827	**4.99E-15**	1.21E-02
rs535586	6p21.33 (31,860,337)	rs12663103	6p21.32 (32,161,324)	5.810	6.66E-11	2.62E-05
rs9267532	6p21.33 (31,639,979)	rs9267487	6p21.33 (31,511,350)	5.806	**6.48E-19**	**3.25E-20**
rs9267487	6p21.33 (31,511,350)	rs9501587	6p21.33 (31,346,937)	5.804	**1.75E-24**	1.96E-05
rs12663103	6p21.32 (32,161,324)	rs3130637	6p21.33 (31,488,145)	5.800	**4.52E-16**	2.79E-05
rs2948369	8p22 (12,736,387)	rs4077920	8q22.1 (98,893,864)	5.800	6.05E-09	1.90E-07

^a^Bold data are genome-wide significant interactions.

### FORCE pinpoints interactions beyond main effects in the HLA region

We also analyzed the exhaustive FORCE results with regard to previous studies, which have detected numerous main effects [24-26], but only few weak statistical interactions [24,34,35]. We assessed the performance of FORCE using the WTCCC psoriasis dataset, which contains 2,618 cases, 2,737 controls and 491,191 SNPs. Table 7 shows the 25 best FORCE hits. Twenty-one out of 25 SNP pairs involve SNPs located in the HLA region on chromosome 6, which is consistent with the known strong involvement of the HLA region in psoriasis. Interestingly, certain SNP pairs found to be statistically significant by FORCE did not reach genome-wide significance when using PLINK FastEpistasis.

It is well known that SNPs with main effects may falsely appear to be interacting [36]. To avoid such artifacts in our analysis, we removed those SNPs that displayed a univariate statistical association P-value of 10^−5^ or less [24]. The results show three highly significant interactions involving SNPs from the HLA region that display no main effect (Table 8). In the absence of correlation between the SNPs we claim that these findings provide evidence of interactive effects involved in psoriasis susceptibility. This confirms that FORCE is able to uncover novel statistical interactions in the HLA region that have not been detected before using conventional approaches.

**Table 8:** **Most significant interactions detected through exhaustive search after main effect SNPs removal**

**rs number**		**Chromosome**		**Position**		**Marginal effect**		**p-value** ^**a**^	**I**_**OR**_	**R** ^**2b**^
						**p-value**				
**SNP1**	**SNP2**	**SNP1**	**SNP2**	**SNP1**	**SNP2**	**SNP1**	**SNP2**			
rs2254556	rs9267532	6	6	31,374,854	31,672,202	0.008	0.076	**1.22E-22**	5.23	0.002
rs9267532	rs2523518	6	6	31,672,202	31,373,351	0.076	0.006	**3.15E-22**	5.15	0.002
rs2596437	rs9267532	6	6	31,371,309	31,672,202	0.006	0.076	**7.56E-22**	5.1	0.002

^a^Bold data are genome-wide significant interactions. ^b^R^2^ were calculated using controls only.

### FORCE systematically detects interactions missed by other approaches

**Figure 1:**
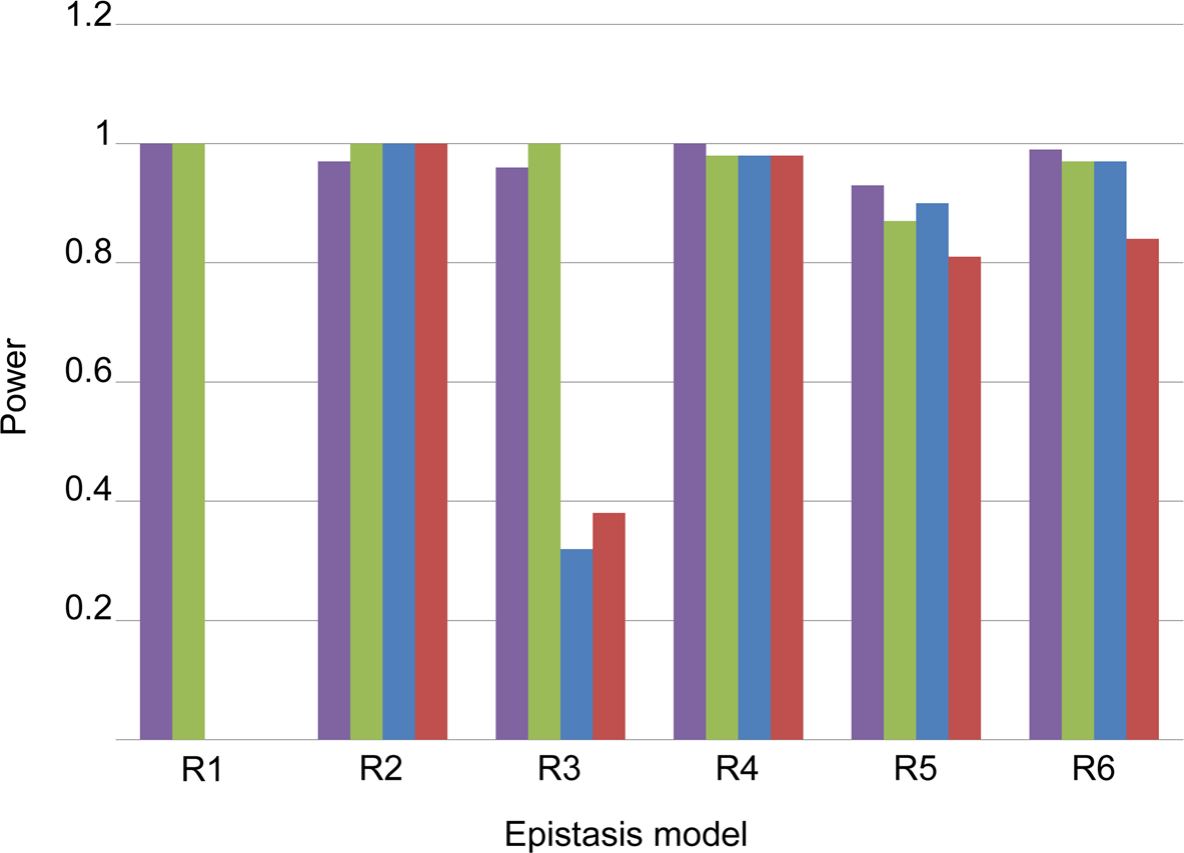
**Power of different approaches to detect simulated epistatic interactions across the six epistasis models by Ritchie [** 28 **].** Purple: FORCE – Green: MB-MDR – Blue: PLINK Epistasis – Red: PLINK FastEpistasis. Refer to Table 3 for the definitions of the 6 interaction models.

Besides its exhaustiveness, the other characteristic feature of the FORCE approach is the use of the I_OR_ statistic for genome-wide epistasis analysis. To study the extent to which the choice of this statistic contributed to the identification of novel statistical interactions, we used datasets that contained different simulated epistatic interactions between SNPs without main effects, according one of six models of Ritchie [28], and none or one of the four sources of noise: Genotyping Error (GE), Missing Data (MS), Genetic Heterogeneity (GH), Phenocopy (PC) (see Methods for details). We then evaluated the power of FORCE and three other popular epistasis detection methods (PLINK Epistasis [7] and PLINK FastEpistasis [8] using default parameters, and MB-MDR [16], using recommended parameters [37]) to detect the simulated interactions. We used a significance threshold of 0.001. Figure 1 shows the results for all epistatic models for the case of no noise.

**Figure 2:**
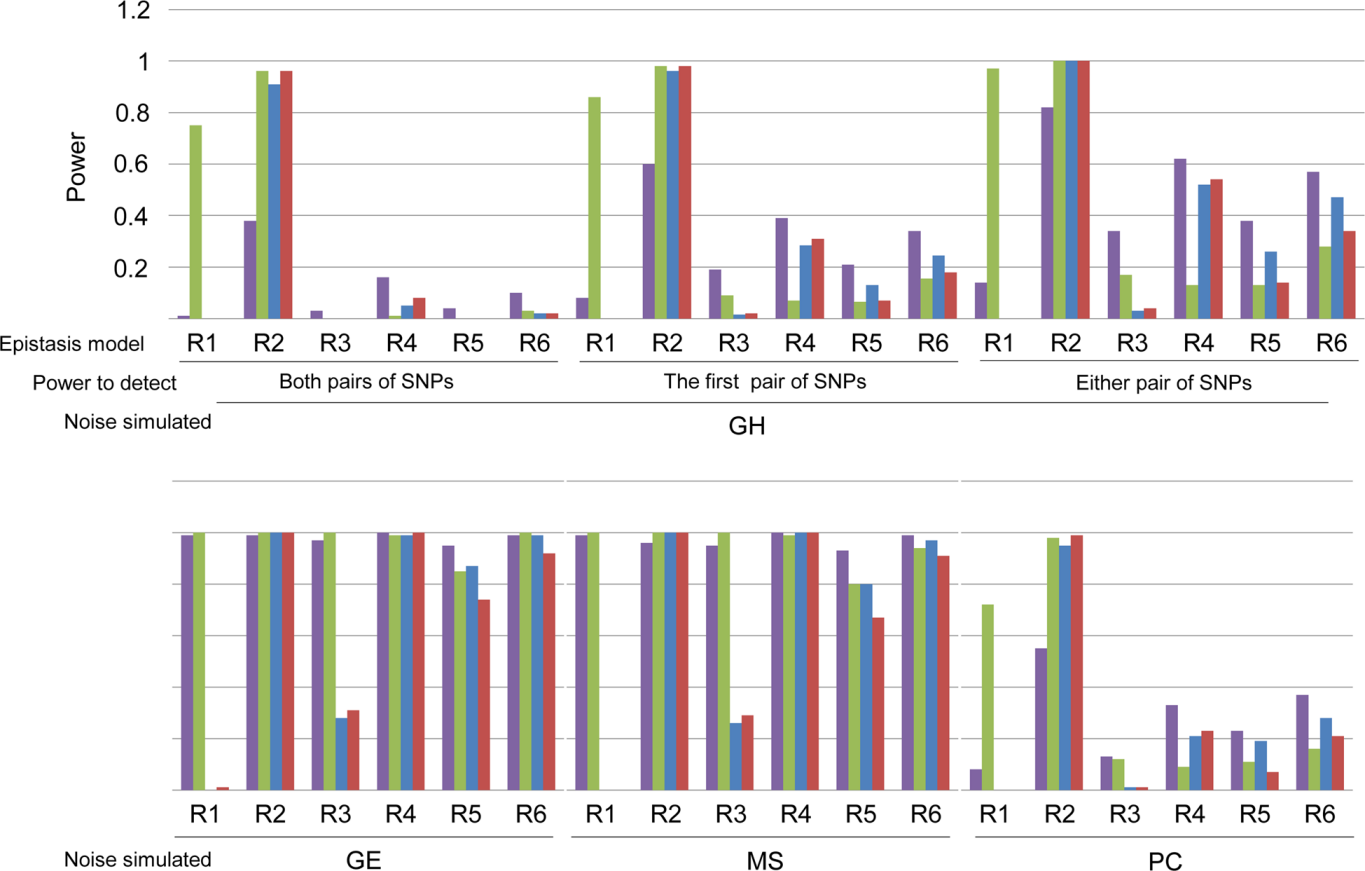
**Power of different approaches to detect simulated epistatic interactions across the six epistasis models by Ritchie [** 28 **], in the presence of noise.** Comparison of the power of four methods to detect interaction in the presence of one source of noise. GH: Genetic heterogeneity – GE: Genotyping errors – MS: Missing data – PC: Phenocopy. When GH is simulated, three different ways of calculating power are employed: the power of detecting both pairs in the same dataset, the power of detecting the first (fixed) pair and the power to detect either of the two epistatic pairs. Purple: FORCE – Green: MB-MDR – Blue: PLINK Epistasis – Red: PLINK FastEpistasis.

Under all six models, FORCE and MB-MDR consistently showed power close to 1. The situation became more interesting in the presence of noise. Figure 2 shows the power of the tested methods for all six models in the presence of one type of noise (numerical values for are given in Tables 9, 10, 11 and 12). While the results for Genotyping Errors (GE) and Missing Data (MS) were very similar to the no-noise scenario, the presence of Genetic Heterogeneity (GH, independent of the definition of “detection”) or Phenocopy (PC) revealed larger differences among the different approaches. Firstly, we noted that, with GH and PC, all approaches lose power. Secondly, we observed that different approaches worked consistently better than others, depending on the interaction model. For interaction models 1 and 2, MB-MDR dominated all other approaches; FORCE dominated the other approaches for interaction models 3–6.

**Table 9:** **Power and family-wise error rate (FWER) of FORCE, MBMDR, Plink Epistasis and Plink FastEpistasis on 6 epistasis models with or without noise**

				**Model 1**	**Model 2**	**Model 3**	**Model 4**	**Model 5**	**Model 6**
**No noise**	**FORCE**		Power^a^	**1**	**0.97**	**0.96**	**1**	**0.93**	**0.99**
			FWER^b^	**0.05**	**0.02**	**0.02**	0.06	**0.05**	**0.04**
	**MBMDR**		Power	**1**	**1**	**1**	**0.98**	**0.87**	**0.97**
			FWER	**0.02**	**0**	**0**	**0.01**	**0.01**	**0.01**
	**Plink**	**Epistasis**	Power	0	**1**	0.32	**0.98**	**0.9**	**0.97**
			FWER	**0.04**	**0.05**	**0.02**	**0.02**	**0**	**0.03**
		**FastEpistasis**	Power	0	**1**	0.38	**0.98**	**0.81**	**0.84**
			FWER	0.07	0.07	**0.02**	**0.02**	**0.01**	**0.05**
**GE**	**FORCE**		Power	**0.99**	**0.99**	**0.97**	**1**	**0.95**	**0.99**
			FWER	**0.03**	**0.03**	0.08	**0.03**	**0.03**	**0.04**
	**MBMDR**		Power	**1**	**1**	**1**	**0.99**	**0.85**	**1**
			FWER	**0**	**0**	**0.01**	**0.02**	**0**	**0**
	**Plink**	**Epistasis**	Power	0	**1**	0.28	**0.99**	**0.87**	**0.99**
			FWER	**0**	**0**	0.07	**0.02**	**0**	**0**
		**FastEpistasis**	Power	0.01	**1**	0.31	**1**	**0.74**	**0.92**
			FWER	**0.04**	**0**	0.09	**0.05**	**0**	**0**
**MS**	**FORCE**		Power	**0.99**	**0.96**	**0.95**	**1**	**0.93**	**0.99**
			FWER	0.07	**0.02**	**0.02**	**0.03**	**0.03**	0.06
	**MBMDR**		Power	**1**	**1**	**1**	**0.99**	**0.8**	**0.94**
			FWER	**0**	**0**	**0**	**0**	**0**	**0**
	**Plink**	**Epistasis**	Power	0	**1**	0.26	**1**	**0.8**	**0.97**
			FWER	**0.03**	**0.02**	0.08	**0.02**	**0**	**0**
		**FastEpistasis**	Power	0	**1**	0.29	**1**	**0.67**	**0.91**
			FWER	0.06	**0.01**	**0.1**	**0.04**	**0**	**0**
**PC**	**FORCE**		Power	0.08	**0.55**	0.13	0.33	0.23	0.37
			FWER	**0.03**	**0.05**	**0.05**	0.06	**0.04**	0.07
	**MBMDR**		Power	**0.72**	**0.98**	0.12	0.09	0.11	0.16
			FWER	**0**	**0**	**0**	**0**	**0**	**0**
	**Plink**	**Epistasis**	Power	0	**0.95**	0.01	0.21	0.19	0.28
			FWER	**0.04**	**0.03**	**0.05**	0.06	**0.01**	**0**
		**FastEpistasis**	Power	0	**0.99**	0.01	0.23	0.07	0.21
			FWER	0.07	**0.03**	**0.04**	0.06	**0.01**	**0.01**

Genotype errors (GE), missing data (MS) or phenocopy (PC). ^a^In bold, power higher than 50%. ^b^In bold, FWER lower than 5%.

**Table 10:** **Power and family-wise error rate (FWER) of FORCE, MBMDR, Plink Epistasis and Plink FastEpistasis on 6 epistasis models without noise or with simulated genetic heterogeneity (GH)**

			**Model 1**			**Model 2**			**Model 3**			**Model 4**			**Model 5**			**Model 6**		
			**Both**	**First**	**Either**	**Both**	**First**	**Either**	**Both**	**First**	**Either**	**Both**	**First**	**Either**	**Both**	**First**	**Either**	**Both**	**First**	**Either**
**FORCE**		Power^a^	0.01	0.08	0.14	0.38	0.6	**0.82**	0.03	0.19	0.34	0.16	0.39	**0.62**	0.04	0.21	0.38	0.1	0.34	**0.57**
		FWER^b^	**0.02**			0.07			0.07			**0.04**			**0.02**			**0.02**		
**MBMDR**		Power	**0.75**	**0.86**	**0.97**	**0.96**	**0.98**	**1**	0	0.09	0.17	0.01	0.07	0.13	0	0.07	0.13	0.03	0.16	0.28
		FWER	**0**			**0**			**0**			**0**			**0**			**0**		
**Plink**	**Epistasis**	Power	0	0	0	**0.91**	**0.96**	**1**	0	0.02	0.03	0.05	0.29	**0.52**	0	0.13	0.26	0.02	0.25	0.47
		FWER	**0.01**			**0.02**			**0.01**			**0.05**			**0**			**0**		
	**FastEpistasis**	Power	0	0	0	**0.96**	**0.98**	**1**	0	0.02	0.04	0.08	0.31	**0.54**	0	0.07	0.14	0.02	0.18	0.34
		FWER	**0.02**			**0.03**			**0.01**			0.07			**0.01**			**0**		

^a^In bold, power higher than 50%. ^b^In bold, FWER lower than 5%.

**Table 11:** **Power of FORCE detection method, impact of various sources of noise and combinations of them for the 6 epistatic models**

**Type of noise**	**Model 1** ^**a**^			**Model 2**			**Model 3**			**Model 4**			**Model 5**			**Model 6**		
No noise	**1**			**0.97**			**0.96**			**1**			**0.93**			**0.99**		
Genotype errors (GE)	**0.99**			**0.99**			**0.97**			**1**			**0.95**			**0.99**		
Phenocopy (PC)	0.08			**0.55**			0.13			0.33			0.23			0.37		
Misssing data (MS)	**0.99**			**0.96**			**0.95**			**1**			**0.93**			**0.99**		
GE + PC	0.05			**0.62**			0.18			0.3			0.31			0.35		
GE + MS	**0.95**			**0.98**			**0.96**			**1**			**0.91**			**0.99**		
PC + MS	0.06			**0.52**			0.21			0.31			0.21			0.26		
GE + PC + MS	0.09			**0.55**			0.21			0.46			0.13			0.35		
	**both**	**first**	**either**	**both**	**first**	**either**	**both**	**first**	**either**	**both**	**first**	**either**	**both**	**first**	**either**	**both**	**first**	**either**
Genetic heterogeneity (GH)	0.01	0.08	0.14	0.38	0.6	**0.82**	0	0.19	0.34	0.2	0.39	**0.62**	0	0.21	0.38	0.1	0.34	**0.57**
GH + GE	0.01	0.09	0.16	0.34	0.6	**0.85**	0.03	0.18	0.32	0.17	0.4	**0.62**	0.04	0.23	0.41	0.14	0.31	**0.50**
GH + PC	0	0.015	0.03	0.02	0.09	0.16	0	0.01	0.02	0	0.02	0.04	0.01	0.035	0.06	0.01	0.04	0.07
GH + MS	0.01	0.04	0.07	0.37	0.57	**0.77**	0.02	0.145	0.27	0.18	0.385	**0.59**	0.03	0.19	0.35	0.07	0.28	**0.50**
GH + GE + PC	0	0.01	0.02	0.03	0.105	0.18	0	0.02	0.04	0	0.025	0.05	0	0.025	0.05	0	0.03	0.06
GH + GE + MS	0	0.05	0.1	0.33	0.665	**0.80**	0.02	0.155	0.29	0.13	0.385	**0.64**	0.03	0.23	0.43	0.13	0.305	0.48
GH + PC + MS	0	0.005	0.01	0.01	0.095	0.18	0	0.025	0.05	0	0.04	0.08	0	0.005	0.01	0	0.035	0.07
GH + GE + PC + MS	0	0.015	0.03	0.01	0.08	0.15	0	0.015	0.03	0	0.035	0.07	0	0.015	0.03	0	0.03	0.06

GE: Genotyping errors – GH: Genetic heterogeneity – MS: Missing data – PC: Phenocopy. In case of GH, power is calculated in 3 different ways as the proportion of datasets in which both, the first or either of the interacting pairs are detected. ^a^In bold, power higher than 50%.

**Table 12:** **Family-wise error rate (FWER) of FORCE for the 6 epistatic models and 16 noise conditions tested**

**Family-wise error rate**	**Model 1** ^**a**^	**Model 2**	**Model 3**	**Model 4**	**Model 5**	**Model 6**
No noise	0.05	0.02	0.02	**0.06**	0.05	0.04
Genotype errors (GE)	0.03	0.03	**0.08**	0.03	0.03	0.04
Genetic heterogeneity (GH)	0.02	**0.07**	**0.07**	0.04	0.02	0.02
Phenocopy (PC)	0.03	0.05	0.05	**0.06**	0.04	**0.07**
Misssing data (MS)	**0.07**	0.02	0.02	0.03	0.03	**0.06**
GE + GH	0.05	**0.07**	0.04	0.03	0.01	**0.07**
GE + PC	0.05	0.05	0.01	0.02	0.03	0.02
GE + MS	0.02	0.01	**0.06**	0.04	0.04	**0.07**
GH + PC	0.05	0.05	0.03	0.02	0.03	0.03
GH + MS	0.04	**0.07**	0.05	0.03	0.03	0.01
PC + MS	0.03	0.02	0.03	**0.06**	0.04	0.03
GE + GH + PC	**0.07**	0.03	**0.06**	0.03	0.02	0.04
GE + GH + MS	**0.07**	0.05	0.04	0.01	0.05	0.05
GH + PC + MS	0.04	0.02	**0.06**	0.02	0.03	0.03
GE + PC + MS	0.05	**0.08**	**0.06**	**0.06**	0.05	0.05
GE + GH + PC + MS	0.02	**0.07**	**0.06**	0.04	0.05	**0.06**

GE: Genotyping errors – GH: Genetic heterogeneity – MS: Missing data – PC: Phenocopy. ^a^In bold, FWER > 0.05.

## Discussion

This study introduces the FORCE approach for genome-wide epistasis analysis. On the basis of the Interaction Odds Ratio (I_OR_) statistic, it performs a genome-wide search for epistatic interactions between pairs of SNPs in a reasonable time on a standard laptop computer. The search is exhaustive and filter-free; i.e., the result is guaranteed to reflect the most extreme I_OR_ values over all possible interactions. Exhaustive search using FORCE is possible because of the computational simplicity of the I_OR_ statistic.

Wu et al. [38] introduced a haplotype-based measure based on the following term:

$$ {I}_{GH}=\frac{OR_{G_1{H}_1}}{OR_{G_1{H}_2}{OR}_{G_2{H}_1}} $$

where ${OR}_{G_1{H}_1}$ is the odds ratio for both risk haplotypes when carried together, compared to the baseline haplotypes; ${OR}_{G_1{H}_2}$ and ${OR}_{G_2{H}_1}$ are the odds ratios for each risk haplotype, respectively, compared to the baseline haplotype.

Although both methods are based on odds ratios, the methods differ in several respects. First, and most significantly, Wu’s method uses haplotypes, which typically require the statistical inference of haplotypes. Even though this design was shown to be better powered than classical genotype-based statistics, the additional calculations are computationally costly. As a result, FORCE can perform an exhaustive genome-wide epistasis search in a few days on a single compute core while, in practice, Wu’s method only allows a limited number of SNP pairs to be tested.

In addition to the different statistics themselves, the approaches to calculating significance differ. FORCE relies on an exact P-value that requires too much time to be calculated exhaustively for all SNP pairs. Instead, P-values are calculated only for pairs with the highest I_OR_. Conversely, Wu et al. used an approximate, chi-square distribution-based, P-value which can be applied to each investigated pair of the search.

Our study on WTCCC psoriasis data suggests that the computational effort for exhaustive testing is currently not just a luxury. The popular class of conditional analyses focuses only on possible interactions of previously implicated SNPs – often the only option to perform large-scale analysis in reasonable time. When comparing conditional and exhaustive FORCE analyses, we found that the conditional approach only detects interactions of vastly weaker statistical significance.

Our systematic study on small simulated datasets indicates that FORCE not only “goes farther” than existing approaches because of its exhaustive search, but also detects fundamentally different types of interactions, in particular in the biologically more relevant models 3–6. In two out of six models of epistatic interaction described by Ritchie [28], and across the different sources of noise in the data, FORCE consistently displayed a good power of detection compared to other approaches. Interestingly, each of the four approaches is always less efficient than another for at least one model associated with one type of noise.

Finally, by applying FORCE to WTCCC psoriasis data, we were able to detect statistical interactions between SNPs in the HLA region, even after the exclusion of all SNPs with main effects. To our knowledge this constitutes the first demonstration that the genetic structure of the HLA region cannot be understood by the analysis of main effects alone and that more than one interacting locus exists in that region.

## Conclusions

Together, the different elements of our study suggest that FORCE represents a valuable new addition to the arsenal of genome-wide epistasis detection approaches for case–control studies. As with other approaches, the additionally detected interactions are *a priori* of a statistical nature, and require detailed analysis and follow-up.

Beyond this, our study has provided an example for the need for exhaustive epistasis analysis. In the future, exhaustive analysis will be facilitated by the ever-increasing computational power available to biological research. On one hand, this may enable the exhaustive calculation of FORCE P-values, which can be expected to lead to a potentially much enlarged set of statistically significant interactions. On the other hand, more computational power, as well as algorithmic improvements, may also render exhaustive analysis under those models of interactions feasible for which running times are prohibitive today. Finally, we believe that these improvements are necessary for the integration of different types of interactions and other types of large-scale data, which may ultimately be key to understanding the genetic basis of complex diseases.

## Additional file

Additional file 1: Table S1. Epistasis analysis among GWAS hits: all 153 pairs of the conditional search. **Table S2.** FORCE Exhaustive search top 100 hits on psoriasis data.

## Abbreviations

OR: Odds ratio
I_OR_: Interaction odds ratio
HLA: Human leukocyte antigen
GWAS: Genome wide association study
SNP: Single nucleotide polymorphism
WTCCC: The welcome trust case control consortium
GE: Genotyping error
MS: Missing data
GH: Genetic heterogeneity
PC: Phenocopy
FWER: Family-wise error rate
