# Supplementary material for: Filter-free exhaustive odds ratio-based genome-wide interaction approach pinpoints evidence for interaction in the HLA region in psoriasis
Source: BMC Genet. 2015 Feb 6;16:11. doi: 10.1186/s12863-015-0174-3 (PMC4341885; doi:10.1186/s12863-015-0174-3)
Supplement: Additional file 1: Table S1. — Epistasis analysis among GWAS hits: all 153 pairs of the conditional search. Table S2. FORCE Exhaustive search top 100 hits on psoriasis data. [file 12863_2015_174_MOESM1_ESM.docx]

**Table S1 - Epistasis analysis among GWAS hits: all 153 pairs**

Results are sorted according to FORCE empirical p-values, estimated on the basis of 10^6^ permutations

| **SNP pair description** | | | | |  | **Epistasis analysis** | | | | |
| --- | --- | --- | --- | --- | --- | --- | --- | --- | --- | --- |
| **First SNP** | |  | **Second SNP** | |  | **FORCE** | |  | **Plink FastEpistasis** | |
| **rs number** | **Chromosomal location** |  | **rs number** | **Chromosomal location** |  | **I_OR_** | **Empirical p-value** |  | **Z-score** | **p-value** |
| rs10484554 | 6p21.33 |  | rs27524 | 5q15 |  | 1.407 | 0.003 |  | 6.846 | 0.009 |
| rs10484554 | 6p21.33 |  | rs3134792 | 6p21.33 |  | 0.676 | 0.008 |  | 1.068 | 0.301 |
| rs2201841 | 1p31.3 |  | rs3213094 | 5q33.3 |  | 1.343 | 0.012 |  | 4.737 | 0.03 |
| rs3134792 | 6p21.33 |  | rs4795067 | 17q11 |  | 1.385 | 0.013 |  | 3.188 | 0.074 |
| rs20541 | 5q31 |  | rs17716942 | 2q24 |  | 0.706 | 0.013 |  | 6.987 | 0.008 |
| rs702873 | 2p16 |  | rs4795067 | 17q11 |  | 0.75 | 0.014 |  | 3.414 | 0.065 |
| rs10484554 | 6p21.33 |  | rs4795067 | 17q11 |  | 0.765 | 0.018 |  | 2.597 | 0.107 |
| rs610604 | 6q23 |  | rs17716942 | 2q24 |  | 0.74 | 0.023 |  | 6.591 | 0.01 |
| rs3213094 | 5q33.3 |  | rs12580100 | 12q13.2 |  | 0.732 | 0.029 |  | 5.132 | 0.023 |
| rs4649203 | 1p36 |  | rs240993 | 6q21 |  | 0.795 | 0.038 |  | 2.27 | 0.132 |
| rs4649203 | 1p36 |  | rs702873 | 2p16 |  | 0.79 | 0.041 |  | 1.237 | 0.266 |
| rs3134792 | 6p21.33 |  | rs27524 | 5q15 |  | 0.767 | 0.041 |  | 11.84 | 5.8E-04 |
| rs702873 | 2p16 |  | rs2546890 | 5q33.3 |  | 0.776 | 0.042 |  | 0.804 | 0.37 |
| rs27524 | 5q15 |  | rs17716942 | 2q24 |  | 0.763 | 0.046 |  | 5.28 | 0.022 |
| rs610604 | 6q23 |  | rs6701216 | 1q21 |  | 0.793 | 0.058 |  | 4.289 | 0.038 |
| rs2201841 | 1p31.3 |  | rs2546890 | 5q33.3 |  | 1.254 | 0.059 |  | 2.587 | 0.108 |
| rs27524 | 5q15 |  | rs7993214 | 13q14.11 |  | 0.807 | 0.06 |  | 3.596 | 0.058 |
| rs3134792 | 6p21.33 |  | rs3213094 | 5q33.3 |  | 1.279 | 0.073 |  | 2.61 | 0.106 |
| rs702873 | 2p16 |  | rs2201841 | 1p31.3 |  | 0.819 | 0.084 |  | 1.669 | 0.196 |
| rs10484554 | 6p21.33 |  | rs12580100 | 12q13.2 |  | 1.259 | 0.087 |  | 2.535 | 0.111 |
| rs4649203 | 1p36 |  | rs6701216 | 1q21 |  | 0.811 | 0.087 |  | 3.518 | 0.061 |
| rs2201841 | 1p31.3 |  | rs27524 | 5q15 |  | 1.212 | 0.089 |  | 1.666 | 0.197 |
| rs4112788 | 1q21.3 |  | rs7993214 | 13q14.11 |  | 0.829 | 0.09 |  | 1.546 | 0.214 |
| rs240993 | 6q21 |  | rs7993214 | 13q14.11 |  | 0.832 | 0.096 |  | 1.896 | 0.169 |
| rs6701216 | 1q21 |  | rs8016947 | 14q13 |  | 1.234 | 0.101 |  | 1.087 | 0.297 |
| rs3213094 | 5q33.3 |  | rs4795067 | 17q11 |  | 0.822 | 0.101 |  | 0.744 | 0.388 |
| rs702873 | 2p16 |  | rs3213094 | 5q33.3 |  | 0.817 | 0.102 |  | 0.899 | 0.343 |
| rs4649203 | 1p36 |  | rs3213094 | 5q33.3 |  | 1.209 | 0.108 |  | 2.623 | 0.105 |
| rs10484554 | 6p21.33 |  | rs702873 | 2p16 |  | 0.832 | 0.112 |  | 0.922 | 0.337 |
| rs240993 | 6q21 |  | rs2546890 | 5q33.3 |  | 0.827 | 0.113 |  | 1.757 | 0.185 |
| rs3134792 | 6p21.33 |  | rs7993214 | 13q14.11 |  | 1.225 | 0.118 |  | 0.46 | 0.497 |
| rs4649203 | 1p36 |  | rs4795067 | 17q11 |  | 1.181 | 0.138 |  | 0.514 | 0.474 |
| rs3213094 | 5q33.3 |  | rs8016947 | 14q13 |  | 0.833 | 0.141 |  | 0.898 | 0.343 |
| rs6701216 | 1q21 |  | rs7993214 | 13q14.11 |  | 1.188 | 0.161 |  | 0.765 | 0.382 |
| rs20541 | 5q31 |  | rs4112788 | 1q21.3 |  | 0.848 | 0.166 |  | 0.584 | 0.445 |
| rs27524 | 5q15 |  | rs6701216 | 1q21 |  | 0.845 | 0.181 |  | 3.074 | 0.08 |
| rs702873 | 2p16 |  | rs7993214 | 13q14.11 |  | 0.857 | 0.183 |  | 0.819 | 0.365 |
| rs2546890 | 5q33.3 |  | rs7993214 | 13q14.11 |  | 0.853 | 0.186 |  | 1.233 | 0.267 |
| rs2546890 | 5q33.3 |  | rs4795067 | 17q11 |  | 0.852 | 0.187 |  | 1.262 | 0.261 |
| rs240993 | 6q21 |  | rs12580100 | 12q13.2 |  | 0.84 | 0.189 |  | 1.647 | 0.199 |
| rs3134792 | 6p21.33 |  | rs702873 | 2p16 |  | 0.841 | 0.199 |  | 0.888 | 0.346 |
| rs27524 | 5q15 |  | rs610604 | 6q23 |  | 0.867 | 0.209 |  | 0.6 | 0.439 |
| rs27524 | 5q15 |  | rs240993 | 6q21 |  | 0.87 | 0.217 |  | 1.955 | 0.162 |
| rs8016947 | 14q13 |  | rs4795067 | 17q11 |  | 1.154 | 0.225 |  | 0.046 | 0.831 |
| rs2546890 | 5q33.3 |  | rs17716942 | 2q24 |  | 1.188 | 0.231 |  | 0.366 | 0.545 |
| rs702873 | 2p16 |  | rs12580100 | 12q13.2 |  | 0.851 | 0.244 |  | 1.447 | 0.229 |
| rs27524 | 5q15 |  | rs20541 | 5q31 |  | 0.869 | 0.25 |  | 0.092 | 0.761 |
| rs4649203 | 1p36 |  | rs3134792 | 6p21.33 |  | 0.865 | 0.262 |  | 1.961 | 0.161 |
| rs3134792 | 6p21.33 |  | rs12580100 | 12q13.2 |  | 0.837 | 0.264 |  | 1.727 | 0.189 |
| rs610604 | 6q23 |  | rs4795067 | 17q11 |  | 1.132 | 0.269 |  | 0.198 | 0.656 |
| rs6701216 | 1q21 |  | rs3213094 | 5q33.3 |  | 1.153 | 0.277 |  | 1.071 | 0.301 |
| rs3134792 | 6p21.33 |  | rs17716942 | 2q24 |  | 0.845 | 0.278 |  | 0.903 | 0.342 |
| rs20541 | 5q31 |  | rs6701216 | 1q21 |  | 1.15 | 0.283 |  | 0.407 | 0.523 |
| rs10484554 | 6p21.33 |  | rs20541 | 5q31 |  | 1.137 | 0.284 |  | 1.304 | 0.254 |
| rs20541 | 5q31 |  | rs4795067 | 17q11 |  | 1.133 | 0.299 |  | 1.701 | 0.192 |
| rs4649203 | 1p36 |  | rs4112788 | 1q21.3 |  | 1.119 | 0.31 |  | 0.653 | 0.419 |
| rs3134792 | 6p21.33 |  | rs2546890 | 5q33.3 |  | 1.149 | 0.318 |  | 0.033 | 0.856 |
| rs4649203 | 1p36 |  | rs20541 | 5q31 |  | 1.126 | 0.322 |  | 0.545 | 0.46 |
| rs27524 | 5q15 |  | rs4795067 | 17q11 |  | 1.12 | 0.324 |  | 0.574 | 0.449 |
| rs702873 | 2p16 |  | rs240993 | 6q21 |  | 0.893 | 0.327 |  | 0.772 | 0.38 |
| rs20541 | 5q31 |  | rs610604 | 6q23 |  | 0.897 | 0.359 |  | 3.094 | 0.079 |
| rs27524 | 5q15 |  | rs12580100 | 12q13.2 |  | 1.131 | 0.369 |  | 1.696 | 0.193 |
| rs240993 | 6q21 |  | rs17716942 | 2q24 |  | 0.89 | 0.379 |  | 0.207 | 0.649 |
| rs12580100 | 12q13.2 |  | rs4795067 | 17q11 |  | 1.124 | 0.384 |  | 0.25 | 0.618 |
| rs10484554 | 6p21.33 |  | rs2546890 | 5q33.3 |  | 1.11 | 0.385 |  | 0.452 | 0.502 |
| rs2201841 | 1p31.3 |  | rs4112788 | 1q21.3 |  | 1.099 | 0.393 |  | 0.435 | 0.509 |
| rs2546890 | 5q33.3 |  | rs8016947 | 14q13 |  | 1.113 | 0.396 |  | 0.045 | 0.832 |
| rs6701216 | 1q21 |  | rs4795067 | 17q11 |  | 1.107 | 0.412 |  | 1.359 | 0.244 |
| rs6701216 | 1q21 |  | rs17716942 | 2q24 |  | 1.127 | 0.413 |  | 0.641 | 0.423 |
| rs3213094 | 5q33.3 |  | rs2546890 | 5q33.3 |  | 0.798 | 0.428 |  | 0 | 0.995 |
| rs17716942 | 2q24 |  | rs7993214 | 13q14.11 |  | 1.11 | 0.432 |  | 0.213 | 0.645 |
| rs3134792 | 6p21.33 |  | rs610604 | 6q23 |  | 0.906 | 0.443 |  | 0.433 | 0.511 |
| rs240993 | 6q21 |  | rs4112788 | 1q21.3 |  | 0.919 | 0.443 |  | 1.383 | 0.24 |
| rs3134792 | 6p21.33 |  | rs240993 | 6q21 |  | 0.908 | 0.454 |  | 2.209 | 0.137 |
| rs17716942 | 2q24 |  | rs12580100 | 12q13.2 |  | 0.887 | 0.456 |  | 0.835 | 0.361 |
| rs4649203 | 1p36 |  | rs12580100 | 12q13.2 |  | 1.103 | 0.461 |  | 0.043 | 0.837 |
| rs610604 | 6q23 |  | rs12580100 | 12q13.2 |  | 0.908 | 0.466 |  | 0.683 | 0.409 |
| rs3134792 | 6p21.33 |  | rs6701216 | 1q21 |  | 0.901 | 0.473 |  | 0.66 | 0.417 |
| rs240993 | 6q21 |  | rs4795067 | 17q11 |  | 1.08 | 0.489 |  | 0.019 | 0.89 |
| rs702873 | 2p16 |  | rs27524 | 5q15 |  | 0.922 | 0.491 |  | 0.349 | 0.555 |
| rs4112788 | 1q21.3 |  | rs12580100 | 12q13.2 |  | 0.913 | 0.495 |  | 0.664 | 0.415 |
| rs3134792 | 6p21.33 |  | rs20541 | 5q31 |  | 0.91 | 0.496 |  | 0.004 | 0.95 |
| rs2201841 | 1p31.3 |  | rs7993214 | 13q14.11 |  | 0.928 | 0.496 |  | 0.448 | 0.503 |
| rs2201841 | 1p31.3 |  | rs4795067 | 17q11 |  | 0.927 | 0.499 |  | 1.656 | 0.198 |
| rs7993214 | 13q14.11 |  | rs4795067 | 17q11 |  | 0.93 | 0.517 |  | 0.191 | 0.662 |
| rs610604 | 6q23 |  | rs4112788 | 1q21.3 |  | 0.932 | 0.526 |  | 0.002 | 0.961 |
| rs10484554 | 6p21.33 |  | rs7993214 | 13q14.11 |  | 0.932 | 0.527 |  | 0.006 | 0.94 |
| rs4112788 | 1q21.3 |  | rs8016947 | 14q13 |  | 0.929 | 0.528 |  | 0.922 | 0.337 |
| rs702873 | 2p16 |  | rs8016947 | 14q13 |  | 0.928 | 0.534 |  | 0.577 | 0.448 |
| rs6701216 | 1q21 |  | rs2546890 | 5q33.3 |  | 0.923 | 0.55 |  | 0.685 | 0.408 |
| rs20541 | 5q31 |  | rs3213094 | 5q33.3 |  | 1.078 | 0.557 |  | 1.249 | 0.264 |
| rs10484554 | 6p21.33 |  | rs6701216 | 1q21 |  | 1.074 | 0.561 |  | 0.13 | 0.719 |
| rs702873 | 2p16 |  | rs6701216 | 1q21 |  | 0.929 | 0.563 |  | 0.001 | 0.973 |
| rs610604 | 6q23 |  | rs8016947 | 14q13 |  | 1.068 | 0.571 |  | 0.61 | 0.435 |
| rs20541 | 5q31 |  | rs12580100 | 12q13.2 |  | 1.081 | 0.584 |  | 0.638 | 0.425 |
| rs702873 | 2p16 |  | rs17716942 | 2q24 |  | 1.079 | 0.588 |  | 0 | 0.997 |
| rs4649203 | 1p36 |  | rs10484554 | 6p21.33 |  | 1.062 | 0.589 |  | 0.198 | 0.656 |
| rs2201841 | 1p31.3 |  | rs17716942 | 2q24 |  | 0.933 | 0.6 |  | 0.031 | 0.86 |
| rs27524 | 5q15 |  | rs2546890 | 5q33.3 |  | 1.063 | 0.617 |  | 0.528 | 0.467 |
| rs610604 | 6q23 |  | rs3213094 | 5q33.3 |  | 1.061 | 0.618 |  | 0.001 | 0.972 |
| rs2201841 | 1p31.3 |  | rs8016947 | 14q13 |  | 0.944 | 0.621 |  | 0.663 | 0.416 |
| rs10484554 | 6p21.33 |  | rs2201841 | 1p31.3 |  | 0.947 | 0.622 |  | 0.002 | 0.966 |
| rs3134792 | 6p21.33 |  | rs4112788 | 1q21.3 |  | 0.939 | 0.626 |  | 0.003 | 0.957 |
| rs4112788 | 1q21.3 |  | rs2546890 | 5q33.3 |  | 1.06 | 0.628 |  | 0.077 | 0.781 |
| rs17716942 | 2q24 |  | rs4795067 | 17q11 |  | 0.937 | 0.631 |  | 0.024 | 0.877 |
| rs4649203 | 1p36 |  | rs610604 | 6q23 |  | 0.949 | 0.635 |  | 0.002 | 0.963 |
| rs4649203 | 1p36 |  | rs2546890 | 5q33.3 |  | 1.058 | 0.638 |  | 0.336 | 0.562 |
| rs702873 | 2p16 |  | rs20541 | 5q31 |  | 0.946 | 0.655 |  | 1.001 | 0.317 |
| rs4649203 | 1p36 |  | rs7993214 | 13q14.11 |  | 1.051 | 0.656 |  | 0.401 | 0.527 |
| rs27524 | 5q15 |  | rs3213094 | 5q33.3 |  | 0.949 | 0.665 |  | 0.006 | 0.938 |
| rs4112788 | 1q21.3 |  | rs17716942 | 2q24 |  | 1.057 | 0.678 |  | 1.417 | 0.234 |
| rs4112788 | 1q21.3 |  | rs3213094 | 5q33.3 |  | 1.05 | 0.678 |  | 0.009 | 0.924 |
| rs240993 | 6q21 |  | rs8016947 | 14q13 |  | 1.049 | 0.679 |  | 0.221 | 0.639 |
| rs6701216 | 1q21 |  | rs12580100 | 12q13.2 |  | 0.941 | 0.682 |  | 0.12 | 0.729 |
| rs610604 | 6q23 |  | rs7993214 | 13q14.11 |  | 0.958 | 0.701 |  | 0.209 | 0.648 |
| rs10484554 | 6p21.33 |  | rs4112788 | 1q21.3 |  | 1.041 | 0.717 |  | 0.013 | 0.908 |
| rs702873 | 2p16 |  | rs4112788 | 1q21.3 |  | 1.043 | 0.718 |  | 1.667 | 0.197 |
| rs4112788 | 1q21.3 |  | rs6701216 | 1q21 |  | 1.045 | 0.72 |  | 0.181 | 0.67 |
| rs12580100 | 12q13.2 |  | rs8016947 | 14q13 |  | 0.951 | 0.721 |  | 0.008 | 0.93 |
| rs4649203 | 1p36 |  | rs8016947 | 14q13 |  | 0.96 | 0.725 |  | 0.063 | 0.802 |
| rs4112788 | 1q21.3 |  | rs4795067 | 17q11 |  | 0.963 | 0.738 |  | 0.018 | 0.895 |
| rs10484554 | 6p21.33 |  | rs610604 | 6q23 |  | 1.037 | 0.741 |  | 0.128 | 0.721 |
| rs4649203 | 1p36 |  | rs27524 | 5q15 |  | 1.037 | 0.75 |  | 1.4 | 0.237 |
| rs2546890 | 5q33.3 |  | rs12580100 | 12q13.2 |  | 1.042 | 0.777 |  | 0.872 | 0.351 |
| rs20541 | 5q31 |  | rs7993214 | 13q14.11 |  | 1.034 | 0.78 |  | 0.087 | 0.768 |
| rs240993 | 6q21 |  | rs3213094 | 5q33.3 |  | 1.033 | 0.78 |  | 0.341 | 0.559 |
| rs702873 | 2p16 |  | rs610604 | 6q23 |  | 0.97 | 0.791 |  | 0.152 | 0.696 |
| rs2201841 | 1p31.3 |  | rs610604 | 6q23 |  | 0.971 | 0.791 |  | 0.001 | 0.973 |
| rs20541 | 5q31 |  | rs2546890 | 5q33.3 |  | 1.032 | 0.806 |  | 0.004 | 0.947 |
| rs240993 | 6q21 |  | rs6701216 | 1q21 |  | 1.029 | 0.812 |  | 0.424 | 0.515 |
| rs4649203 | 1p36 |  | rs2201841 | 1p31.3 |  | 0.976 | 0.825 |  | 0.21 | 0.647 |
| rs3134792 | 6p21.33 |  | rs2201841 | 1p31.3 |  | 0.973 | 0.834 |  | 1.913 | 0.167 |
| rs27524 | 5q15 |  | rs4112788 | 1q21.3 |  | 0.979 | 0.85 |  | 0.014 | 0.908 |
| rs27524 | 5q15 |  | rs8016947 | 14q13 |  | 0.978 | 0.854 |  | 0.063 | 0.802 |
| rs2201841 | 1p31.3 |  | rs240993 | 6q21 |  | 0.982 | 0.865 |  | 0.004 | 0.947 |
| rs610604 | 6q23 |  | rs2546890 | 5q33.3 |  | 1.019 | 0.874 |  | 0.561 | 0.454 |
| rs4649203 | 1p36 |  | rs17716942 | 2q24 |  | 1.021 | 0.874 |  | 0.168 | 0.682 |
| rs12580100 | 12q13.2 |  | rs7993214 | 13q14.11 |  | 1.021 | 0.876 |  | 0.522 | 0.47 |
| rs3213094 | 5q33.3 |  | rs17716942 | 2q24 |  | 1.022 | 0.878 |  | 0.052 | 0.82 |
| rs3134792 | 6p21.33 |  | rs8016947 | 14q13 |  | 0.984 | 0.905 |  | 0.048 | 0.826 |
| rs10484554 | 6p21.33 |  | rs17716942 | 2q24 |  | 1.015 | 0.91 |  | 2.3E-04 | 0.988 |
| rs17716942 | 2q24 |  | rs8016947 | 14q13 |  | 0.988 | 0.932 |  | 0.049 | 0.824 |
| rs3213094 | 5q33.3 |  | rs7993214 | 13q14.11 |  | 0.99 | 0.933 |  | 0.176 | 0.675 |
| rs240993 | 6q21 |  | rs20541 | 5q31 |  | 0.99 | 0.934 |  | 0.076 | 0.783 |
| rs2201841 | 1p31.3 |  | rs20541 | 5q31 |  | 1.009 | 0.942 |  | 0.073 | 0.788 |
| rs7993214 | 13q14.11 |  | rs8016947 | 14q13 |  | 0.993 | 0.951 |  | 0.001 | 0.975 |
| rs10484554 | 6p21.33 |  | rs8016947 | 14q13 |  | 0.993 | 0.953 |  | 0.253 | 0.615 |
| rs2201841 | 1p31.3 |  | rs12580100 | 12q13.2 |  | 1.007 | 0.96 |  | 0.098 | 0.755 |
| rs20541 | 5q31 |  | rs8016947 | 14q13 |  | 0.996 | 0.975 |  | 0.037 | 0.847 |
| rs10484554 | 6p21.33 |  | rs3213094 | 5q33.3 |  | 1.003 | 0.98 |  | 0.003 | 0.955 |
| rs2201841 | 1p31.3 |  | rs6701216 | 1q21 |  | 0.998 | 0.985 |  | 0.088 | 0.767 |
| rs10484554 | 6p21.33 |  | rs240993 | 6q21 |  | 0.999 | 0.991 |  | 0.312 | 0.576 |
| rs240993 | 6q21 |  | rs610604 | 6q23 |  | 1.001 | 0.994 |  | 0.158 | 0.691 |
|  |  |  |  |  |  |  |  |  |  |  |

**Table S2 - FORCE Exhaustive search top 100 hits on psoriasis data**

| **First SNP** | |  | **Second SNP** | |  | **Exhaustive search results** | |
| --- | --- | --- | --- | --- | --- | --- | --- |
| **rs number** | **Chromosomal location** |  | **rs number** | **Chromosomal location** |  | **u(I_OR_)** | **p-value** |
| rs4151664 | 6p21.33 |  | rs9267532 | 6p21.33 |  | 10.588 | 3.32E-33 |
| rs4151664 | 6p21.33 |  | rs2227956 | 6p21.33 |  | 9.662 | 2.02E-26 |
| rs3132468 | 6p21.33 |  | rs4151664 | 6p21.33 |  | 9.571 | 3.14E-25 |
| rs9267546 | 6p21.33 |  | rs4151664 | 6p21.33 |  | 8.34 | 1.08E-31 |
| rs4151664 | 6p21.33 |  | rs2260000 | 6p21.33 |  | 7.749 | 3.74E-18 |
| rs2523608 | 6p21.33 |  | rs4151664 | 6p21.33 |  | 7.695 | 1.08E-18 |
| rs4151664 | 6p21.33 |  | rs2855807 | 6p21.33 |  | 7.444 | 3.88E-17 |
| rs2596464 | 6p21.33 |  | rs4151664 | 6p21.33 |  | 7.379 | 2.67E-15 |
| rs3129939 | 6p21.32 |  | rs3131296 | 6p21.32 |  | 7.376 | 6.43E-41 |
| rs2516464 | 6p21.33 |  | rs12663103 | 6p21.32 |  | 7.229 | 4.25E-13 |
| rs6906662 | 6p21.32 |  | rs9267649 | 6p21.33 |  | 7.187 | 1.59E-25 |
| rs12153855 | 6p21.33 |  | rs2523608 | 6p21.33 |  | 7.181 | 1.59E-23 |
| rs4149013 | 12p12.2 |  | rs9356206 | 6q27 |  | 6.485 | 9.82E-09 |
| rs535586 | 6p21.33 |  | rs2523589 | 6p21.33 |  | 6.299 | 1.84E-44 |
| rs2523589 | 6p21.33 |  | rs659445 | 6p21.33 |  | 6.268 | 4.08E-45 |
| rs408359 | 6p21.32 |  | rs4151664 | 6p21.33 |  | 6.038 | 4.30E-21 |
| rs2164182 | 11q21 |  | rs16864296 | 1q24.3 |  | 5.945 | 8.34E-08 |
| rs2227956 | 6p21.33 |  | rs2523589 | 6p21.33 |  | 5.851 | 1.39E-42 |
| rs12050395 | 14q31.3 |  | rs2301092 | 5q14.3 |  | 5.831 | 1.67E-08 |
| rs12663103 | 6p21.32 |  | rs9267649 | 6p21.33 |  | 5.827 | 4.99E-15 |
| rs535586 | 6p21.33 |  | rs12663103 | 6p21.32 |  | 5.81 | 6.66E-11 |
| rs9267532 | 6p21.33 |  | rs9267487 | 6p21.33 |  | 5.806 | 6.48E-19 |
| rs9267487 | 6p21.33 |  | rs9501587 | 6p21.33 |  | 5.804 | 1.75E-24 |
| rs12663103 | 6p21.32 |  | rs3130637 | 6p21.33 |  | 5.800 | 4.52E-16 |
| rs2948369 | 8p22 |  | rs4077920 | 8q22.1 |  | 5.800 | 6.05E-09 |
| rs6440853 | 3q25.2 |  | rs7595804 | 2p25.1 |  | 5.796 | 4.07E-08 |
| rs644827 | 6p21.33 |  | rs6906662 | 6p21.32 |  | 5.796 | 1.77E-14 |
| rs3093993 | 6p21.33 |  | rs2523589 | 6p21.33 |  | 5.792 | 2.68E-43 |
| rs2523589 | 6p21.33 |  | rs3130637 | 6p21.33 |  | 5.775 | 3.58E-43 |
| rs9267532 | 6p21.33 |  | rs499691 | 6p21.32 |  | 5.772 | 1.51E-25 |
| rs2517532 | 6p21.33 |  | rs12663103 | 6p21.32 |  | 5.767 | 3.72E-11 |
| rs2523589 | 6p21.33 |  | rs3095227 | 6p21.33 |  | 5.74 | 6.58E-43 |
| rs6906662 | 6p21.32 |  | rs3130637 | 6p21.33 |  | 5.727 | 6.60E-21 |
| rs7770216 | 6p21.33 |  | rs9267649 | 6p21.33 |  | 5.704 | 6.78E-49 |
| rs11569523 | 19p13.3 |  | rs17268268 | 13q32.1 |  | 5.702 | 1.24E-08 |
| rs408359 | 6p21.32 |  | rs9267487 | 6p21.33 |  | 5.685 | 6.99E-20 |
| rs7356880 | 6p21.32 |  | rs535586 | 6p21.33 |  | 5.684 | 3.14E-11 |
| rs3093993 | 6p21.33 |  | rs12663103 | 6p21.32 |  | 5.681 | 1.02E-15 |
| rs2596464 | 6p21.33 |  | rs3830041 | 6p21.32 |  | 5.676 | 2.89E-16 |
| rs2523589 | 6p21.33 |  | rs9267649 | 6p21.33 |  | 5.673 | 8.98E-41 |
| rs12663103 | 6p21.32 |  | rs3095227 | 6p21.33 |  | 5.666 | 1.10E-15 |
| rs4151664 | 6p21.33 |  | rs13437082 | 6p21.33 |  | 5.665 | 8.97E-18 |
| rs9267649 | 6p21.33 |  | rs9266395 | 6p21.33 |  | 5.66 | 2.46E-48 |
| rs4711269 | 6p21.33 |  | rs4151664 | 6p21.33 |  | 5.647 | 1.01E-17 |
| rs3093993 | 6p21.33 |  | rs6906662 | 6p21.32 |  | 5.637 | 1.53E-20 |
| rs4711268 | 6p21.33 |  | rs4151664 | 6p21.33 |  | 5.637 | 1.11E-17 |
| rs9262632 | 6p21.33 |  | rs4151664 | 6p21.33 |  | 5.634 | 3.46E-15 |
| rs9267649 | 6p21.33 |  | rs6933050 | 6p21.33 |  | 5.626 | 3.61E-48 |
| rs6906662 | 6p21.32 |  | rs3095227 | 6p21.33 |  | 5.622 | 1.71E-20 |
| rs6906662 | 6p21.32 |  | rs2596464 | 6p21.33 |  | 5.59 | 4.40E-13 |
| rs9266409 | 6p21.33 |  | rs9267649 | 6p21.33 |  | 5.559 | 1.59E-47 |
| rs2227956 | 6p21.33 |  | rs12663103 | 6p21.32 |  | 5.554 | 1.79E-14 |
| rs3093662 | 6p21.33 |  | rs3132468 | 6p21.33 |  | 5.548 | 1.17E-24 |
| rs9378200 | 6p21.33 |  | rs535586 | 6p21.33 |  | 5.548 | 6.22E-17 |
| rs659445 | 6p21.33 |  | rs7356880 | 6p21.32 |  | 5.529 | 2.45E-11 |
| rs3093662 | 6p21.33 |  | rs408359 | 6p21.32 |  | 5.527 | 1.59E-20 |
| rs727108 | 17q21.32 |  | rs11785015 | 8p21.1 |  | 5.521 | 4.22E-08 |
| rs6906662 | 6p21.32 |  | rs3132468 | 6p21.33 |  | 5.512 | 4.66E-18 |
| rs12663103 | 6p21.32 |  | rs659445 | 6p21.33 |  | 5.51 | 6.47E-11 |
| rs7356880 | 6p21.32 |  | rs6932730 | 6p21.33 |  | 5.508 | 1.95E-15 |
| rs659445 | 6p21.33 |  | rs9378200 | 6p21.33 |  | 5.506 | 2.54E-17 |
| rs2596464 | 6p21.33 |  | rs9267487 | 6p21.33 |  | 5.487 | 2.30E-15 |
| rs9267487 | 6p21.33 |  | rs3869129 | 6p21.33 |  | 5.484 | 6.34E-22 |
| rs3130637 | 6p21.33 |  | rs2844480 | 6p21.33 |  | 5.482 | 6.10E-51 |
| rs3130931 | 6p21.33 |  | rs12663103 | 6p21.32 |  | 5.472 | 8.77E-14 |
| rs2227956 | 6p21.33 |  | rs7770216 | 6p21.33 |  | 5.454 | 7.18E-47 |
| rs9469003 | 6p21.33 |  | rs6933050 | 6p21.33 |  | 5.435 | 2.53E-42 |
| rs2229094 | 6p21.33 |  | rs6906662 | 6p21.32 |  | 5.42 | 3.75E-20 |
| rs2260000 | 6p21.33 |  | rs12663103 | 6p21.32 |  | 5.411 | 2.03E-12 |
| rs9266395 | 6p21.33 |  | rs2227956 | 6p21.33 |  | 5.41 | 2.62E-46 |
| rs6933050 | 6p21.33 |  | rs9501106 | 6p21.33 |  | 5.404 | 4.15E-42 |
| rs9501106 | 6p21.33 |  | rs7770216 | 6p21.33 |  | 5.395 | 5.11E-42 |
| rs9501106 | 6p21.33 |  | rs9266409 | 6p21.33 |  | 5.392 | 6.12E-42 |
| rs4151664 | 6p21.33 |  | rs2516464 | 6p21.33 |  | 5.39 | 3.21E-09 |
| rs9267487 | 6p21.33 |  | rs9267649 | 6p21.33 |  | 5.382 | 1.10E-24 |
| rs7770216 | 6p21.33 |  | rs9469003 | 6p21.33 |  | 5.375 | 8.01E-42 |
| rs9378200 | 6p21.33 |  | rs4151664 | 6p21.33 |  | 5.372 | 3.76E-15 |
| rs17567779 | 5q13.3 |  | rs981705 | 7p21.1 |  | 5.365 | 7.32E-08 |
| rs3129939 | 6p21.32 |  | rs4151664 | 6p21.33 |  | 5.365 | 8.77E-22 |
| rs6933050 | 6p21.33 |  | rs2227956 | 6p21.33 |  | 5.36 | 6.21E-46 |
| rs9266409 | 6p21.33 |  | rs9469003 | 6p21.33 |  | 5.358 | 1.32E-41 |
| rs3093662 | 6p21.33 |  | rs2523608 | 6p21.33 |  | 5.356 | 2.02E-13 |
| rs2596464 | 6p21.33 |  | rs7356880 | 6p21.32 |  | 5.347 | 2.48E-09 |
| rs204994 | 6p21.32 |  | rs6906662 | 6p21.32 |  | 5.346 | 6.35E-18 |
| rs2227956 | 6p21.33 |  | rs9267487 | 6p21.33 |  | 5.344 | 1.89E-24 |
| rs9266845 | 6p21.33 |  | rs7356880 | 6p21.32 |  | 5.341 | 2.90E-15 |
| rs10209151 | 2p25.1 |  | rs10272549 | 7p15.2 |  | 5.339 | 9.48E-08 |
| rs2516464 | 6p21.33 |  | rs4947324 | 6p21.33 |  | 5.323 | 4.82E-16 |
| rs2227956 | 6p21.33 |  | rs9266409 | 6p21.33 |  | 5.317 | 1.56E-45 |
| rs733980 | 14q31.3 |  | rs1396374 | 11q14.3 |  | 5.277 | 6.85E-09 |
| rs3093998 | 6p21.33 |  | rs6906662 | 6p21.32 |  | 5.274 | 2.61E-16 |
| rs12663103 | 6p21.32 |  | rs3132468 | 6p21.33 |  | 5.27 | 1.28E-13 |
| rs9266395 | 6p21.33 |  | rs9501106 | 6p21.33 |  | 5.263 | 1.08E-40 |
| rs4626906 | 1p21.1 |  | rs1521015 | 5p14.2 |  | 5.259 | 1.28E-07 |
| rs2069662 | 5q13.3 |  | rs1438270 | 14q31.1 |  | 5.256 | 2.42E-08 |
| rs7356880 | 6p21.32 |  | rs1131904 | 6p21.33 |  | 5.254 | 4.84E-15 |
| rs9267487 | 6p21.33 |  | rs659445 | 6p21.33 |  | 5.253 | 1.13E-13 |
| rs9469003 | 6p21.33 |  | rs9266395 | 6p21.33 |  | 5.244 | 1.68E-40 |
| rs9266825 | 6p21.33 |  | rs7356880 | 6p21.32 |  | 5.243 | 5.96E-15 |
| rs9501106 | 6p21.33 |  | rs3093662 | 6p21.33 |  | 5.239 | 8.56E-24 |
|  |  |  |  |  |  |  |  |
